# Graphene-Based Ammonia Sensors Functionalised with Sub-Monolayer V_2_O_5_: A Comparative Study of Chemical Vapour Deposited and Epitaxial Graphene †

**DOI:** 10.3390/s19040951

**Published:** 2019-02-23

**Authors:** Margus Kodu, Artjom Berholts, Tauno Kahro, Jens Eriksson, Rositsa Yakimova, Tea Avarmaa, Indrek Renge, Harry Alles, Raivo Jaaniso

**Affiliations:** 1 Institute of Physics, University of Tartu, W. Ostwald Street 1, EE50411 Tartu, Estonia; artjom.berholts@ut.ee (A.B.); tauno.kahro@ut.ee (T.K.); tea.avarmaa@ut.ee (T.A.); indrek.renge@ut.ee (I.R.); harry.alles@ut.ee (H.A.); raivo.jaaniso@ut.ee (R.J.); 2 Department of Physics, Chemistry and Biology, Linköping University, 58183 Linköping, Sweden; jens.eriksson@liu.se (J.E.); rositsa.yakimova@liu.se (R.Y.)

**Keywords:** ammonia, CVD graphene, epitaxial graphene on SiC, gas sensor, pulsed laser deposition, selectivity, single layer graphene, UV light activation, vanadium (V) oxide

## Abstract

Graphene in its pristine form has demonstrated a gas detection ability in an inert carrier gas. For practical use in ambient atmosphere, its sensor properties should be enhanced with functionalisation by defects and dopants, or by decoration with nanophases of metals or/and metal oxides. Excellent sensor behaviour was found for two types of single layer graphenes: grown by chemical vapour deposition (CVD) and transferred onto oxidized silicon (Si/SiO_2_/CVDG), and the epitaxial graphene grown on SiC (SiC/EG). Both graphene samples were functionalised using a pulsed laser deposited (PLD) thin V_2_O_5_ layer of average thickness ≈ 0.6 nm. According to the Raman spectra, the SiC/EG has a remarkable resistance against structural damage under the laser deposition conditions. By contrast, the PLD process readily induces defects in CVD graphene. Both sensors showed remarkable and selective sensing of NH_3_ gas in terms of response amplitude and speed, as well as recovery rate. SiC/EG showed a response that was an order of magnitude larger as compared to similarly functionalised CVDG sensor (295% vs. 31% for 100 ppm NH_3_). The adsorption site properties are assigned to deposited V_2_O_5_ nanophase, being similar for both sensors, rather than (defect) graphene itself. The substantially larger response of SiC/EG sensor is probably the result of the smaller initial free charge carrier doping in EG.

## 1. Introduction 

Graphene, as an atomically thin (semi)conducting material, is a promising sensor platform for monitoring key environmental pollutants, such as NO_x_, NH_3_, SO_2_, CO, H_2_S, O_3_, volatile organic compounds (VOCs), etc. The extraordinary sensitivity of graphene conductance down to a single adsorbed molecule has been demonstrated in the inert gas atmosphere [1]. Graphene has the great potential for device miniaturisation, low-power operation at the room temperature, and low production cost. Achieving good sensing properties under real atmospheric conditions with high background concentration of oxygen and water vapour has remained a challenge. A large amount of research deals with the improvement of graphene-based gas sensors, in particular their sensitivity, selectivity, stability, and speed of response and recovery. These requirements to any viable sensor can be memorized as a “4-S” principle. Selective enhancement of gas adsorption, and therefore, sensor properties, is possible via the introduction of defects or dopants into the graphene lattice [2,3]. Alternatively, precious metals [4,5,6] and semiconducting metal oxides [6,7,8,9] in the form of nanoparticles or thin layers were grown on the graphene with the aim to improve its gas sensing characteristics. 

For graphene functionalisation, pulsed laser deposition (PLD) is recommended as a highly versatile thin-film deposition technique with a relatively well-controlled synthesis process. It is possible to evaporate practically any solid with a focused laser pulse, thus providing the broadest possible selection of materials for various applications [10]. By changing the inert gas background pressure, the kinetic energy of particles is tuned between 0.1 and 1000 eV. The amount of deposited material, starting from only about 1/100^th^ of a monolayer per laser pulse, is also worth mentioning as a benefit of PLD.

A single-layer, chemical vapour deposited (CVD) graphene, modified with PLD of ZrO_2_ and Ag nanostructured coatings shows substantially improved sensing of NO_2_ [6]. High sensitivity with respect to highly oxidizing, free radical NO_2_ gas is nearly universal. Both CVD graphene transferred to insulating substrate [11] and epitaxial graphene on SiC [12,13] are capable of detecting NO_2_ gas down to a few ppb in synthetic air, and thus applicable as NO_2_ environmental sensors.

Proper functionalisation is needed for selectivity towards less reactive polluting gases. Vanadium (V) oxide is known to be an efficient NH_3_ adsorber, widely used for catalytic removal of NO_x_ from exhaust gases in the reactions with sacrificial NH_3_ [14]. Indeed, a sub-nanometer layer of V_2_O_5_, deposited using PLD, renders the CVD graphene a sensitive and selective ammonia gas sensor [15].

The present study will address further important aspects of NH_3_ sensing. Bearing in mind commercially applicable sensors, the properties of graphene, their substrates, and connecting electrode materials, among other features, should be considered. Miniature sensors can be made by transferring the CVD graphene sheet onto functional sensor platforms, equipped with a built-in microheater or embedded light source for better control of operating conditions. Epitaxial graphene on SiC could also be valuable due to a simpler manufacturing process. Moreover, SiC/EG has a higher sensitivity to chemical doping because the intrinsic carrier concentration is less [16]. In this work, the two graphenes of very different origin will be considered in parallel. First, functionalisation of CVD graphene and epitaxial graphene grown on SiC substrate with a few layers of laser deposited V_2_O_5_ is carried out. Raman spectroscopy is applied to reveal the effect of PLD on defect creation in graphene. The influence of functionalisation on NH_3_ gas sensing properties is studied by comparing pristine and V_2_O_5_ deposited graphenes. Second, the gas response measurements are performed in the dark and under ultraviolet light exposure. Also, the dependence of sensor response characteristics on varying humidity level is investigated. Responses of both types of graphene sensors to other polluting gases NO_2_, CO, SO_2_ and O_3_ were tested for comparison in order to assess selectivity. Sensor response and recovery curves are approximated to double exponential functions. Tentative mechanisms of elementary processes are proposed based on phenomenological, biphasic kinetics.

A short version of this paper has appeared in conference proceedings [17].

## 2. Materials and Methods

**Figure 1:**
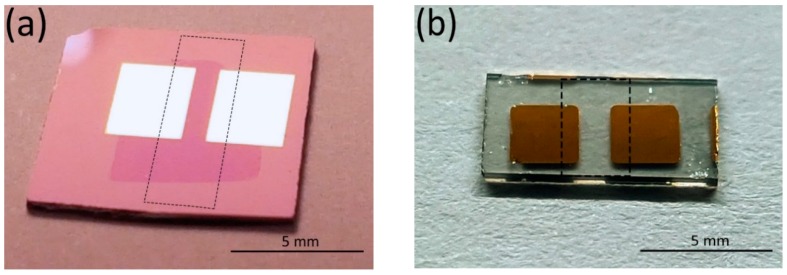
Photographs of gas sensor devices based on (**a**) PLD-functionalised CVD graphene on a Si/SiO_2_ substrate, and (**b**) epitaxial graphene on a SiC substrate. Gaps between the electrodes are 1 × 4 mm^2^ and 1 × 2 mm^2^ for CVD and epitaxial graphene, respectively. The CVD graphene sheet on top of electrodes appears as a darker pink area in (a) due to slightly different reflection properties. The areas of laser deposition of V_2_O_5_ are marked with dashed lines.

Chemical vapour deposited (CVD) graphene (CVDG) was grown on a commercial, 25-µm-thick polycrystalline copper foil in a home-built CVD reactor. The graphene film was transferred onto a Si/SiO_2_ substrate carrying Au (60 nm) electrodes, deposited through a shadow mask via magnetron sputtering (Figure 1a). The graphene growth and transfer process is already thoroughly described in [15].

Large area epitaxial graphene (EG) was grown using a sublimation method on Si-terminated 4H-SiC (0001) substrates at 2000 °C in argon gas at a pressure of 1 bar [18]. Au (200 nm) contact pads were made using DC-sputtering on top of SiC/EG substrate.

In the PLD process, which is specified in [15], a ceramic V_2_O_5_ target was ablated using a KrF excimer laser at wavelength 248 nm and laser pulse energy density of 5.0 J/cm^2^ in the presence of 5 × 10^−2^ mbar of O_2_. A total of 120 laser pulses was used for V_2_O_5_ layer deposition. Sensor substrates were kept at the room temperature during deposition. For comparison, another V_2_O_5_ layer was deposited onto a fused quartz substrate while keeping the experimental conditions exactly the same so that in the following, the mass thickness of deposited layer can be evaluated in the x-ray fluorescence (XRF) measurement.

The CVD graphene was probed using a micro-Raman spectroscopic system (Renishaw, inVia, Gloucestershire, UK) at the excitation wavelength of 514 nm, with a spot diameter of ≈1 μm and incident radiant power of 1 mW. In case of epitaxial SiC graphene 17 mW of 532 nm laser light was focussed to a spot with diameter of ≈0.9 μm. For the V_2_O_5_ decorated epitaxial graphene sample, the laser power was reduced to 1 mW in order to diminish the risk of surface damage. The Raman spectrum of graphene on SiC was obtained after subtracting a reference Raman spectrum of pure carrier material 4H-SiC (0001) from the graphene spectrum.

The amount of vanadium in the deposited layer was assessed with X-ray fluorescence method, and the oxidation state of deposited vanadium was determined by X-ray photoelectron spectroscopy, as described earlier [15].

Gas sensitivity was measured at room temperature in a 7 cm^3^ stainless steel sample chamber, equipped with a gas mixing system, which is more thoroughly described in [19]. The voltage applied to the electrodes was either 100 mV or 500 mV in the cases of CVD graphene and epitaxial graphene samples, respectively. The neat gases or mixtures used in our measurements were N_2_, O_2_, CO/N_2_, NO_2_/N_2_, SO_2_/N_2_, O_2_/N_2_ and NH_3_/N_2_, all 99.999% pure. All used gases and mixtures were of certified composition from AGA (The Linde Group, Estonian branch, Tallinn, Estonia). A gas mixture of 10% O_2_ + 90% N_2_ was flown through a UV lamp ozoniser for O_3_ production, and the resulting O_3_ concentration was monitored using an ozone analyser (model 430, Teledyne API, San Diego, CA, USA). The gas flow through the sample chamber was kept constant at 200 sccm. The nominal relative humidity of the testing gas was regulated between 0 and 50% during the measurements. During the experiments with UV light excitation, the 365 nm light intensity on the sample was ≈15 mW/cm^2^.

## 3. Results

### 3.1. Characterization of Sensor Material Structure and Composition

**Figure 2:**
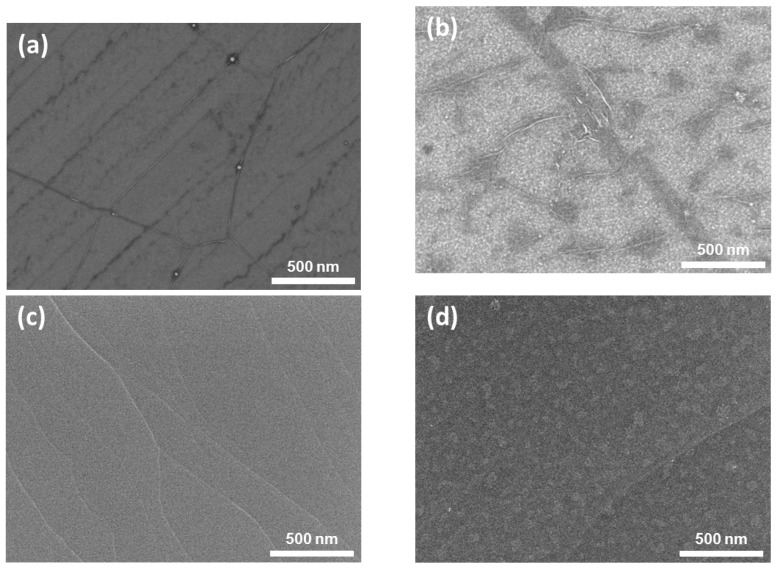
SEM images of pristine and PLD-treated graphene surfaces: (**a**) pristine CVD graphene, (**b**) CVD graphene functionalised with V_2_O_5_, (**c**) pristine epitaxial graphene on SiC and (**d**) the same functionalised with V_2_O_5_.

Scanning electron microscope (SEM) images are displayed in Figure 2 for CVD and epitaxial graphene surfaces before and after the PLD of V_2_O_5_. The features appearing darker in the SEM of CVD graphene (Figure 2a,b) are typical for transferred CVD graphene. It seems that graphene follows the uneven surface topography of polycrystalline Cu foil used in the synthesis; some of the details are possibly wrinkles formed during the graphene transfer. The surface of pristine epitaxial graphene sample in Figure 2c is typical for graphene grown on a nominally single crystalline, on-axis, 4H-SiC substrate. It is characterized by wide terraces, which are due to the step-bunching of the SiC substrate surface that occurs during the high temperature growth [20]. Laser deposited nanostructured material could be distinguished in the SEM pictures of CVD (Figure 2b) and epitaxial (Figure 2d) graphene samples as numerous irregular dots and patches.

**Figure 3:**
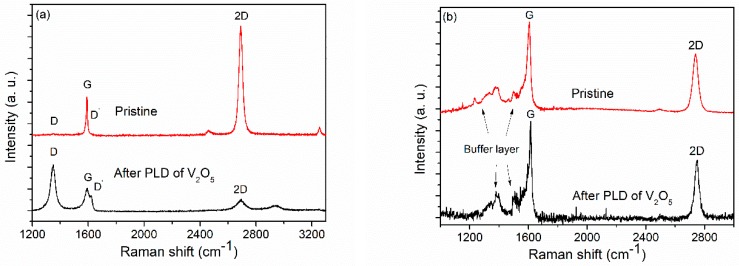
(**a**) Typical Raman spectra of CVD graphene in sensor device, recorded between the electrodes before and after laser deposition of V_2_O_5_: strong defect related peaks D and D’ emerge after deposition of V_2_O_5_. (**b**) Raman spectra of SiC/EG before and after deposition of V_2_O_5_. Lower signal to noise ratio in the lower trace was due to diminished laser power by a factor of 17. Defect related peaks D and D’ were not created noticeably in case of SiC/EG/V_2_O_5_.

The Raman scattering cross section of graphene was remarkably large, owing to phenomena related to resonance enhancement, and was highly useful for structure characterisation. In the case of CVD graphene, the thick supporting material Si/SiO_2_ contributed surprisingly little to scattering intensity, so that the graphene spectrum could easily be separated. Figure 3a displays a typical Raman spectrum of pristine CVD graphene, recorded in the gap between the electrodes of the gas sensor setup. The G and 2D band peaks at ≈1590 cm^−1^ and ≈2690 cm^−1^, and have full-width at half-maximum (FWHM) of 11 cm^−1^ and 29 cm^−1^, respectively. These parameters, together with the 2D to G peak intensity ratio of approximately one to three, corresponded to a single layer of graphene [21]. Missing the defect-related D peak, usually located at ≈1350 cm^−1^, is an indication of low defect density in the pristine CVD graphene sample [21,22]. However, following PLD of V_2_O_5_ onto graphene, prominent defect-related D and D´ bands emerged in the Raman spectrum, and, at the same time, the G and 2D bands decreased in height (see Figure 3a).

Figure 3b compares Raman spectra for the as-grown epitaxial graphene (SiC/EG) and the same decorated with V_2_O_5_. The scattering onset beginning at about 1280 cm^−1^ and extending into the G-peak region originates from the interfacial “buffer” layer between the graphene and SiC substrate, and despite overlap with the expected D peak, is not related to defects [23]. Since these features extended up to the position of the G peak, they also gave rise to an apparent increase in G peak intensity, so that the G line appeared to have a higher intensity than the 2D band, even for monolayer graphene on SiC [8].

Remarkably, for EG on a SiC substrate, the typical Raman peaks indicated that graphene remained intact after the standard PLD treatment. The only noticeable difference between the spectra was higher signal to noise ratio in SiC/EG/V_2_O_5_ due to the lower laser power used, and a slight narrowing and blue-shift of the G and 2D peaks, which can originate from several factors influencing the Raman bands of SiC/EG. Mechanical strain was the major cause affecting the positions of characteristic epitaxial graphene Raman peaks [24], compared to exfoliated graphene. Strain inhomogeneity is recognized for causing variations of peak positions and widths, even in the Raman spectra taken from the same sample [25,26]. Change in graphene doping levels as a result of graphene-substrate interaction is known to influence the positions and widths of G and 2D bands of SiC/EG samples [24,27]. Charge transfer can also take place during graphene functionalisation with V_2_O_5_. The FWHM of the 2D peak in SiC/EG with V_2_O_5_ was 34.5 cm^−1^, which is characteristic for monolayer graphene. 

The laser ablation plasma species have a broad kinetic energy distribution. A noticeable portion of these particles may have energy sufficient for creating point defects as a result of removal of C atoms from the graphene lattice (20 to 100 eV) [28]. As a consequence of laser deposition of V_2_O_5_ onto CVD graphene, the Raman lines assignable to point defects or imperfect graphene edges emerged in the spectrum (Figure 3a). However, according to the Raman spectra depicted in Figure 3b, there were no noticeable defect related lines, and thus no considerable increase of defectiveness in the SiC/EG/V_2_O_5_ sample.

The Raman spectrum of graphene and graphene related systems was fairly sensitive to structural disorder. The ratio of the D and G peaks *I*_D_/*I*_G_ characterizes disorder induced by point defects [22,29,30]. However, the relationship between *I*_D_/*I*_G_ and average distance between the point defects *L*_D_ is non-monotonic and switches from a *I*(D)/*I*(G) ∝ *L*_D_^2^ type of dependence to a *I*(D)/*I*(G) ∝ 1/*L*_D_^2^ type at around *L*_D_ ≈ 4 nm [30]. Because of the increased width of the G and D´ peaks of spectrum in Figure 3a, it is safe to conclude that we were in the region where *L*_D_ < 4 nm. From the ratio *I*_D_/*I*_G_ = 2.88, obtained from Figure 3a, the formulae given by Ferrari et al. [22] yields the average distance between point defects as being *L*_D_ ≈ 2.3 nm, and the defect density *n*_D_ of graphene as ≈6.1 × 10^12^ cm^−2^. Therefore, the initially pristine CVD graphene with very low defect density suffers damage during the PLD process, acquiring a high level of structural disorder. By contrast, according to Raman analysis, the PLD process did not noticeably increase the defectiveness of the epitaxial graphene sample. The conclusions about the influence of PLD reached on the basis of Raman spectra were supported by the electrical conductivity of samples. The conductivity of the CVD graphene sample, which had the Raman spectrum depicted in Figure 3a, decreased by 4 times after the PLD process, plausibly because of the decreased charge carrier mobility of the defective graphene lattice. The conductivity of the epitaxial graphene sample was unchanged after the PLD, which implies much less of a destructive influence.

The amount and oxidation state of vanadium deposited with exactly the same PLD procedure was investigated in our previous work by means of X-ray fluorescence (XRF) and X-ray photoelectron spectroscopy (XPS) [15]. In accordance with the vanadium XPS spectrum and the measured mass thickness of deposited V, the material deposited on graphene was predominately V_2_O_5_ with an average thickness of about 0.6 nm.

### 3.2. The Role of Sensor Functionalisation

**Figure 4:**
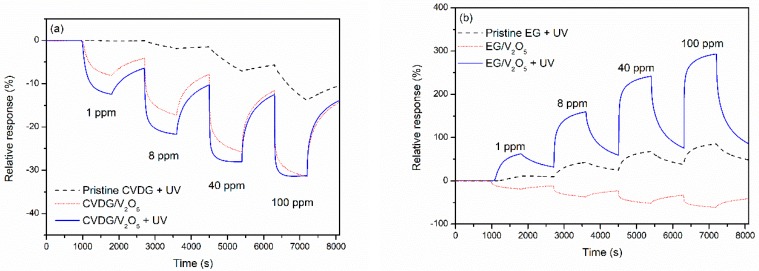
Electric conduction response of sensors based on V_2_O_5_ functionalised (**a**) CVD graphene on Si/SiO_2_, and (**b**) EG on SiC, to varying NH_3_ concentrations at the room temperature. Results for pristine graphene are shown as a reference. Permanent UV light was on during the measurements, except for a single dark run shown for comparison. Relative humidity was 20%. All time intervals of gas exposure and recovery are 900 s.

All sensor measurements in this work were done at room temperature (293–298 K), mainly under continuous excitation with ultraviolet (UV) light (λ = 365 nm), except for several experiments performed without UV exposure for comparison. The influence of graphene functionalisation on the response to NH_3_ gas is illustrated for CVD and epitaxial graphene sensors in Figure 4. The response is defined as a relative change of conductance *G*: *S* = (*G* − *G*_0_)/*G*_0_, where *G*_0_ is the conductance in synthetic air.

The conductivity of CVD graphene and epitaxial graphene sensors changes to the opposite direction after introducing NH_3_ into the test chamber. This was in accordance with the most prominent difference between Si/SiO_2_/CVDG and SiC/EG given their doping pattern in terms of majority charge carriers, p and n, respectively. In general, CVD graphene exhibits p-type conductivity and gas response at normal atmospheric conditions, primarily because of chemical doping by adsorbed oxygen and water molecules [31,32], and possibly, due to interaction with ≡Si–O–H groups on the oxidised silicon substrate. In the case of epitaxial graphene grown on the Si face of 4H-SiC (0001), however, electrons are most often the major carriers, because of charge transfer from the SiC substrate [33,34]. Bearing in mind that NH_3_ acts as a hole acceptor (electron donor) with respect to pristine graphene [1], the conductivity of CVD graphene is expected to decrease, and that of epitaxial graphene to increase, in accordance with observations. A different behaviour of EG without UV light (see the red curve in Figure 4b) is discussed in the next Section 3.3.

The electrical response dynamics of a 2D chemiresistor follows the kinetics of adsorption and chemical processes taking place on the surface. In general, the time dependent gas response to NH_3_ and the subsequent recovery curves can be suitably fitted with double exponential functions that give much better approximation than single exponentials:

(1) $$G\left(t\right)=G_{0}+A_{1}\left[1-e^{\frac{-\left(t-t_{0}\right)}{t_{1}}}\right]+A_{2}\left[1-e^{\frac{-\left(t-t_{0}\right)}{t_{2}}}\right],$$

in Equation (1), *G*_0_ is the initial conductance and *G*(*t*) is the conductance at time *t*; and *t*_0_ is the initial moment of time, when the respective stepwise change in gas composition is introduced. The time-independent coefficients *A*_1_ and *A*_2_ are amplitudes of conductance change. Characteristic times *t*_1_ and *t*_2_ are inverse first-order rate constants for adsorption and desorption kinetics of gas molecules for pristine or V_2_O_5_ functionalised graphene [35].

In principle, a single exponential response corresponds to a single type of adsorption site available for molecules in the surface of graphene sensor. In contrast, the double exponential response (Equation (1)) would describe the situation where two sites with different adsorption and desorption rate constants exist [35]. In addition, double-exponential response and recovery kinetics may originate from competitive adsorption of different gas species, besides NH_3_. For instance, H_2_O and O_2_ are known to readily adsorb on graphene surface and influence the electrical properties of graphene [31,32].

Baseline of the sensor signal (that is, conductivity in synthetic air at some definite relative humidity level) is extremely stable. However, after exposing the sensor to some analyte gas, signal recovery to the baseline can take quite a long time, even when UV light excitation is used. This can be seen in Figure 4, where the sensor signal does not relax completely back to the baseline before the next NH_3_ gas injection. This was because the signal relaxation was not exponential but tended to be double-exponential, whereas one of the exponents had quite a large time constant. The reasons behind this slow recovery can be several and depend on the exact mechanism behind the sensor response: high desorption energy barrier of NH_3_ molecule, or slow re-oxidation of V_2_O_4_ to V_2_O_5_, or some slow charge balancing processes between graphene and substrate etc.

A slow response and recovery of the graphene-based gas senor signal is quite common in the literature [1,3,4,8]. It depends on the practical task at hand, but generally, the fast sensor response and recovery is preferred in applications. However, preliminary experiments in our lab have shown that there can be certain graphene/functionalising material combinations in which case the gas responses and recoveries can be considerably faster. In addition, using short heat pulses during sensor work or doping with precious metals and nanoparticles are other possibilities to speed up the processes.

The results depicted in Figure 4 and Table 1 and Table 2 clearly show that, as compared to the pristine sensors, the response to NH_3_ was considerably improved after functionalisation using PLD. The relative response amplitudes to 8 ppm NH_3_, under UV light excitation, increased from 5 to 22%, and from 50 to 160% for CVDG and EG samples, respectively. Further, much faster characteristic response times were measured on both types of sensors (Table 1 lines 1 and 4, Table 2 lines 1 and 4), but the improvement of response and recovery dynamics was more drastic for the CVDG sensor. The increase of the gas response amplitude probably arose from increased density of energetically favourable adsorption sites or/and increased charge transfer between the graphene/V_2_O_5_ system and NH_3_ adsorbate molecules [15]. Such an improvement can arise from the strong adsorption ability of NH_3_ on V_2_O_5_ and possible redox reactions on the surface of V_2_O_5_ [36,37]. Similarly, fairly good NH_3_ gas sensing properties of semiconducting V_2_O_5_ thin films have been demonstrated by Huotari et al. [38].

### 3.3. Effect of UV Excitation

Simultaneous exposure to UV light can enhance the sensing performance of graphene-based gas sensors drastically. By using UV irradiation, a considerably quicker response to tested gases, and also a faster recovery of the signal, has been reported [11,19,39]. This effect is likely a consequence of cleaning the surface from interfering or passivating gases. The results have been rationalised as photoinduced desorption of oxygen and water molecules, thereby vacating additional adsorption sites on graphene for the target gas. The effect of adsorption activation can be seen in the case of a CVD graphene sensor at lower gas concentrations where the response time and response amplitude improve considerably under UV light illumination (Figure 4a and Table 1). The recovery time of the CVDG sensor also shortened to some extent due to increased desorption.

In case of the EG sensor, the effect of UV light was particularly drastic, as the response to NH_3_ gas without UV light was characteristic of a p-type sensor (i.e., the conductivity decreased upon adding NH_3_), whilst it changed to the n-type response under the UV light. Previously, a switch from an n- to p-type response has been observed for epitaxial graphene gas sensors under increasing concentrations of NO_2_ [40]. The effect was explained in terms of withdrawal of electrons from EG by adsorption of electron-accepting molecular dopant NO_2_, which results in a lower concentration of free electrons, making the holes a major charge carrier. Besides water vapour and O_2_, which act as p-type dopants when adsorbing onto graphene in the ambient air [31,32], there may be yet undetermined adsorbing gases capable of strongly p-doping both the transferred CVD graphene and epitaxial graphene on SiC [41,42]. Therefore, the p-type response of an EG sensor sample is probably caused by adsorbed water vapour, O_2_ and other molecules, which can chemically p-dope graphene and cause n- to p-type switching of pristine graphene, as well as the EG/SiC/V_2_O_5_ system. As discussed before, the EG functionalisation with V_2_O_5_ did not influence the conductivity and defectiveness of epitaxial graphene samples, and is, therefore, probably not accompanied by a major change in electronic properties (mobility and concentration of charge carriers) of epitaxial graphene. Under the UV light exposure, the photo-activated desorption of molecules responsible for p-doping results in epitaxial graphene exhibiting n-type conductivity.

### 3.4. Influence of Humidity

**Figure 5:**
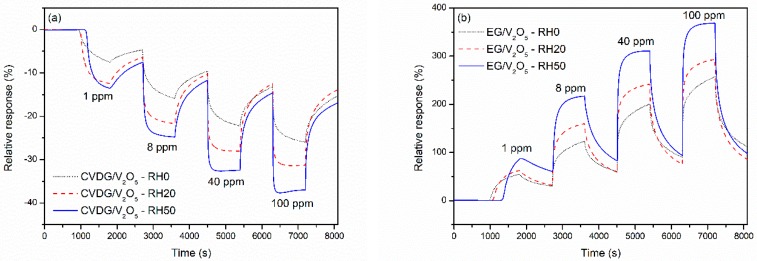
Influence of humidity content on gas responses of sensors based on V_2_O_5_ functionalised (**a**) CVD graphene on Si/SiO_2_, and (**b**) EG on SiC to varying NH_3_ concentrations at the room temperature. Permanent UV light was on during the measurements; the time intervals of gas exposure and recovery were 900 s.

In order to clarify the possible effect of varying humidity level on NH_3_ gas sensing properties, the measurements were carried out under variable relative humidity (RH%) levels of RH0, RH20 and RH50. The results are depicted in Figure 5. Clearly, the response time and amplitude improved with the increasing humidity level for both CVD and epitaxial graphene sensors. Under RH50, as compared to RH0 conditions, the characteristic response time *t*_2_ at 8 ppm NH_3_ decreased by 2.1 and 2.6 times for CVDG and EG sensors, respectively. At the same time, the response amplitude increased from 16% to 25% for CVDG, and from 131% to 216% for EG sensor. This clearly implies an important role of H_2_O in the NH_3_ adsorption mechanism on the surface of sensing layer.

The adsorption of ammonia on V_2_O_5_ has been thoroughly investigated to improve the catalytic reduction of NO_2_ in the presence of NH_3_, an important technology for detoxification of internal combustion engines exhausts. Two strongly bound species are typically observed as a result of the reaction of NH_3_ with V_2_O_5_ adsorption sites: one with the surface OH group (Brønsted acid site), forming a positively charged NH_4_^+^, and the other with the oxygen vacancy (Lewis acid site), forming a species, which is denoted as “coordinated NH_3_” [43]. Although a theoretical study conducted by Yin et al. [44] indicates that adsorption of NH_3_ takes place at both sites, adsorption at the Brønsted site is probably energetically more favourable than adsorption at Lewis site. According to Lin et al. [45], the number of Brønsted surface sites on V_2_O_5_ is directly related to the presence of water vapor. As a consequence of increasing H_2_O concentration in the system, a number of Lewis adsorption sites are converted to Brønsted sites (surface OH groups) by H_2_O adsorption on Lewis sites [45]. Therefore, a faster and larger response under humid measuring conditions of functionalised graphene sensor indicates the favourable role of Brønsted-type adsorption sites over the Lewis-type sites in V_2_O_5_.

### 3.5. Langmuir Model Fitting of Sensor Response

**Figure 6:**
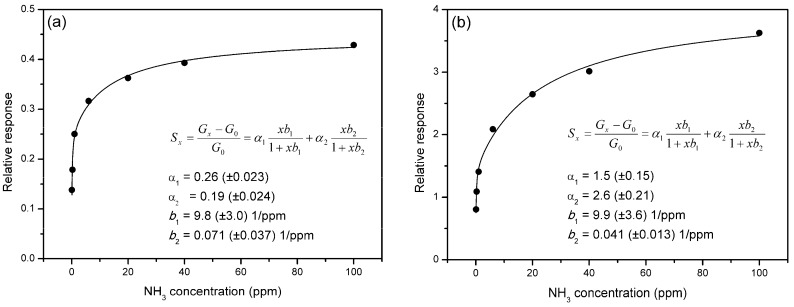
Dependence of stationary relative response amplitudes on NH_3_ concentration (black dots) for (**a**) CVD graphene and (**b**) epitaxial graphene sensors functionalised with V_2_O_5_. The continuous lines are fitting curves to two-site Langmuir adsorption model (Equation (2)). The coefficients of the modelled curves are also shown.

In Figure 6, the stationary relative response of graphene sensors functionalised with V_2_O_5_ is plotted against NH_3_ concentration between 0.1 and 100 ppm. The response to NH_3_ gas in this concentration range was between 0.14 and 0.43 for the CVDG sensor and between 0.80 and 3.63 for the EG sensor. The dependence of responses on NH_3_ concentration followed a Langmuir-type ratiometric function. However, it turned out that stationary responses could not be fitted with a single-site Langmuir adsorption model, but closely followed a two-site Langmuir model [35]:

(2) $$S_{x}=\frac{G_{x}-G_{0}}{G_{0}}=\alpha_{1}\frac{x\cdot b_{1}}{1+x\cdot b_{1}}+\alpha_{2}\frac{x\cdot b_{2}}{1+x\cdot b_{2}},$$

where *b*_1_ and *b*_2_ are the affinity constants, and α_1_ and α_2_ are transduction coefficients, according to the simplified transducer function model for CVD graphene oxygen sensor in [35]. Of course, Equation (2) presumes that transduction coefficients do not depend on the surface coverage, i.e., the change of carrier density and mobility per unit change of coverage does not depend on coverage itself, assuming no screening effects or interactions between adsorbates [35]. The Langmuir function is applicable in the case of a homogenous surface with a single type of adsorption site with a definite adsorption energy. In this case, only the first half of Equation (2) is needed to describe the system. If one assumes the existence of two sites for gas adsorption with different energetic parameters, the model leads to Equation (2), which was used for fitting the experimental data. Fitting of data points in Figure 6 yielded the following values of parameters for the CVDG sensor: α_1_ = 0.26 (±0.023), α_2_ = 0.19 (±0.024), *b*_1_ = 9.8 (±3.0) 1/ppm, *b*_2_ = 0.071 (±0.037) 1/ppm; and the following parameters for the EG sensor: α_1_ = 1.5 (±0.15), α_2_ = 2.6 (±0.21), *b*_1_ = 9.9 (±3.6) 1/ppm, *b*_2_ = 0.041 (±0.013) 1/ppm.

Here, two important aspects are to be noted:

(a) The transduction coefficients α_1_ and α_2_ were both much smaller in case of the CVD graphene sensor, which means either a smaller influence of gas adsorption on the conductivity, or a lower concentration of total active adsorption sites.
(b) The values of corresponding affinity constants *b*_1_ and *b*_2_ were very close for both types of graphene sensors, which mean that rate constants for adsorption and desorption were similar for both types of functionalised graphene.

The point (a) is discussed further below. The last deduction is also clearly evident from data in Table 1 and Table 2 where it can be seen that characteristic times for the response and recovery were nearly the same for functionalised graphene sensors. This means that the type of defects and their adsorption energies were almost the same in the case of functionalised CVD and epitaxial graphene. In addition, the ratios α_1_/α_2_ amounted to 1.4 and 0.58 for CVDG and EG sensors, respectively, which means that the proportion of high affinity sites was considerably higher in the case of the CVD graphene or that their influence on the sensor’s conductivity was proportionally lower in the case of the EG sensor.

### 3.6. Sensor Selectivity

In sensors built on a single layer graphene and other graphene-related materials, the sensitivity to strongly oxidizing NO_2_ gas is generally much higher than that to other gases from the list of important gaseous pollutants. Strong binding and large charge transfer undoubtedly accompany NO_2_ molecule adsorption on either pristine, highly defective, or doped graphene [2,3]. Still, theoretical considerations indicate that the reducing NH_3_ molecule can also interact strongly with defects or dopants in graphene, and this is confirmed to a certain extent by experimental evidence [3].

**Figure 7:**
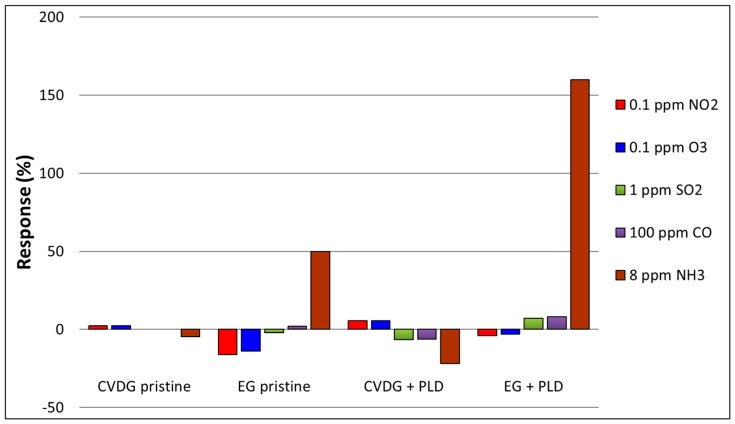
Comparison of the response amplitudes to different polluting gases of CVD graphene and epitaxial graphene sensors with and without laser deposited V_2_O_5_ layer.

The responses of our CVD and epitaxial graphene sensor devices to several polluting gases are compared in Figure 7. We have already shown previously [15] that single layer CVD graphene functionalised with laser deposited V_2_O_5_ was considerably more selective to NH_3_ gas, rather than to NO_2_. PLD functionalisation of CVD graphene with Ag or ZrO_2_, in the contrary, resulted in a higher selective response towards NO_2_ gas [15]. The same tendency is also seen in Figure 7. Compared to the pristine sensors of both types of graphene, the response to NH_3_ gas increased considerably through functionalisation, and at the same time, the response to NO_2_ gas diminished, as in the case of EG, or increased only slightly, as in the case of CVDG. 

The functionalisation with V_2_O_5_ also enhanced the response to CO and SO_2_ gases for both types of sensors. Reactions to O_3_ and NO_2_ gases were quite similar, which was expected, as both molecules are strongly oxidizing, with electron affinity (EA) of 2.1028 eV and 2.273 eV [46], respectively.

## 4. Discussion

Pulsed laser deposition (PLD) was used to create a sub-nanometer V_2_O_5_ layer on graphene samples, either synthesized using chemical vapour deposition (CVD graphene) and transferred to Si/SiO_2_, or epitaxial graphene on a SiC substrate. Raman analysis showed that the defectiveness of CVD graphene increased drastically following PLD, which is an expected result of bombardment with a high energy PLD plasma species. By contrast, the defect concentration of epitaxial graphene is not affected by an identical PLD process, which implies considerable resilience against defect creation. This finding is in accordance with the fact that the electrical conductivity of the SiC/EG sample did not change after the PLD of V_2_O_5_, while the conductivity of CVD graphene was substantially reduced. 

As a possible explanation, we refer to self-healing phenomenon, i.e., reknitting of holes in the graphene during annealing at elevated temperatures [47], or even at room temperature under scanning electron beam [48]. Chen et al. [47] reported the partial recovery of Raman peaks and conductivity of the damaged graphene after annealing at 300 °C. We have also noticed partial recovery of the structure and conductivity of PLD treated CVD graphene samples after keeping them in vacuum at 150 °C. By contrast, at room temperature, the healing process is unlikely, resulting in severe defect creation in case of CVD graphene on Si/SiO_2_ substrate subject to PLD plasma.

Since the self-healing does not occur at 295 K, the negligible deterioration of structure and conductivity in the epitaxial graphene could rather mean considerably higher energy threshold for defect creation. The substrate supporting graphene can play a major role under a flux of highly energetic particles (≈1 MeV), in which case the majority of defects found in graphene are created using the backscattered ions or sputtered atoms from the substrate [49]. For impinging particles having considerably lower energy (up to 10 keV), the indirect defect creation mechanism may still predominate over direct damage in the case of supported graphene [50]. The damage threshold energy was much higher for supported graphene, as compared to suspended graphene [49,50].

Bearing in mind a much tighter graphene-to-support contact, resilience to plasma treatment of SiC/EG can be understood. The transition layer between the sp^2^ graphene and the sp^3^ bulk material in SiC/EG is covalently bound to SiC substrate and serves as a template during epitaxial growth of graphene on the Si face of SiC [20]. Thus, every atom of growing graphene is in an optimal position, and the graphene closely follows the substrate surface topography. On the other hand, CVDG transferred onto the Si/SiO_2_ surface can be quasi-suspended, with the van der Waals contact area probably much less than 100%. In other words, the potential for C atoms in epi-graphene has a much deeper minimum. Thus, in qualitative terms, SiC/EG is more durable under the bombardment with high velocity particles, owing to the stronger bond of the graphene sheet with the underling substrate.

A large enhancement of response to the reducing NH_3_ gas was achieved for both types of graphene sensors as a result of laser-deposited, thin V_2_O_5_ functionalising layer. The magnitude of the relative response of SiC/EG/V_2_O_5_ sensor was particularly noteworthy, for instance, amounting to 310% for 40 ppm NH_3_ at 50% relative humidity. The stationary gas response to the lowest NH_3_ concentration that could be tested, 0.1 ppm, was 14% and 80% at 20% relative humidity for CVDG and SiC/EG, respectively. 

Perfect graphene is relatively inert with respect to chemisorption due to the negligible amount of dangling bonds and a lack of charged atoms on the surface [2,3]. Pristine graphene samples always contain, often unidentified, dopant atoms, defects and organic residues left from the polymer film. Some of these species may even be beneficial when considering the gas adsorption ability. The defects or dopant atoms in the graphene lattice, or atom clusters on graphene surface, can promote adsorption of gaseous pollutants. The effect of adsorption on electric characteristics is also modified [7,51]. Some gas sensitivity, usually in conjunction with very slow sensor recovery at room temperature, is characteristic of pristine graphene, indicating the presence of binding sites with high adsorption energy, occurring at a low density in CVD [3], epitaxial [4], and exfoliated graphene [1]. During functionalisation using PLD, a high number of point defects is formed by bombardment with high energy atoms in the case of CVD graphene. More importantly, the system is modified by adding functionalising oxide material [6,15]. Basing on results shown in Figure 4 and data in Table 1 and Table 2, an increased response and faster dynamics was obvious for functionalised sensors. Functionalised graphene has a higher density of gas adsorption sites with suitable binding energies, resulting in a larger and faster sensor response.

The response dependence on NH_3_ concentration is well approximated using a two-site Langmuir model, which yielded two affinity constants *b*_1_ and *b*_2_, with a difference by two orders of magnitude in both graphenes. However, the corresponding affinities were similar in both instances, showing that CVDG/V_2_O_5_ and SiC/EG/V_2_O_5_ sensors have sites with similar adsorption energies, i.e., the adsorption sites belonged to the functionalising V_2_O_5_ layer, and did not depend on the type of the graphene. The comparable NH_3_ adsorption affinities of both types of graphene would produce similar responses and recovery times of sensors (Table 1 and Table 2).

We were able to advance only tentative explanations about the elementary mechanisms, based on NH_3_ adsorption and conversion processes on V_2_O_5_ surface that are quite extensively covered in the literature on heterogeneous catalysis (see, for example, [36,37]). Among our main findings is the biphasic response kinetics and the amplitude enhancement in the presence of humidity. The possible surface redox reactions taking place in case of graphene/V_2_O_5_ sensor were previously discussed in [15]. Broadly speaking, V^5+^ was perhaps reduced to V^4+^ by ammonia and the polaron thus formed migrated in the V_2_O_5_ lattice and finally reached graphene. The experiments potentially allowing one to distinguish between different mechanisms of graphene conductivity change, as well as to establish the chemical reactions between NH_3_ and O_2_ on the surface are in progress.

As can be observed form the measurement results in Figure 5 and Figure 7, both pristine and functionalised epitaxial graphene gas sensors had nearly ten times higher relative response to NH_3_ gas than the corresponding CVD graphene sensors. This was reflected in the transduction coefficients α_1_ and α_2_ in the Langmuir model (Equation (2)), which were both much smaller in the case of CVD graphene. The major part of the more sensitive nature of EG was most probably related to the particular feature of gas sensing mechanism of the graphene. It was found by Schedin et al. [1] that charge transfer is the main mechanism of gas sensitivity in the case of exfoliated graphene sheet placed on top of the Si/SiO_2_ substrate, e.g., the gas adsorption solely modified the concentration of free charge carriers in graphene without changing their mobility. If we assume the same for our sensors, then the change of carrier mobility, caused by the NH_3_ adsorption was smaller than the effect of carrier concentration, and the gas response amplitude *S* is determined by a relative change of charge carrier numbers Δ*n*/*n*_0_ [35]:

(3) $$S=\frac{G-G_{0}}{G_{0}}=\frac{\tilde{n}\cdot \Delta \theta }{n_{0}},$$

where Δθ is the fractional coverage change for the adsorbed species, *n*_0_ is the carrier concentration in the absence of adsorbate gas, and the constant $\tilde{n}$ characterises the change of carrier density per unit change of coverage.

The amount of charge transferred per unit change of coverage (i.e., density of adsorption sites and charge transferred per site) was similar for both types of graphene sensors. It follows that the lower initial concentration of carriers *n*_0_ of the graphene would correspond to a stronger gas response, and vice versa. In sensors used in this work, the conductivity of pristine CVD graphene was about 15 times higher than that for epitaxial graphene. Given that typical carrier mobility is not very different for pristine samples of CVDG and EG, 1000–3000 and ≈1000 cm^2^·V^−1^·s^−1^, respectively, the charge carrier density in CVDG should be about one order of magnitude higher than that in EG. This conclusion is well supported by the literature data on the typical electronic doping levels of SiC/EG and Si/SiO_2_/CVDG.

Both epitaxial graphene grown on Si-terminated 4H-SiC (0001) and CVD graphene transferred to Si/SiO_2_ are typically highly doped, owing to interactions with the underlying substrate or surface adsorbates, i.e., water, oxygen and other adsorbate molecules [41,42]. Room temperature charge carrier concentration, due to doping effects are typically near 10^12^ cm^−2^ in the case of SiC/EG [41,52,53] and near 10^13^ cm^−2^ in the case of Si/SiO_2_/CVDG [42]. Since adsorption sites seem to be energetically similar for both types of sensors, the difference in the response magnitudes to NH_3_ (by an order of magnitude) probably arose as a result of the lower initial doping level of SiC/EG. In other words, the Fermi energy level being closer to the Dirac point allowed for higher sensitivity to chemical doping, induced by adsorbing NH_3_ molecules. The proximity of the Fermi level to a Dirac point in SiC/EG was supported by the fact that under UV light, which is capable of removing the major atmospheric dopants, the response of EG-based sensors switched from p- to n-type.

## 5. Conclusions

The functionalisation of CVD-grown and epitaxial graphene with V_2_O_5_ enhanced the response and improved the selectivity of chemiresistive sensors with respect to ammonia. The analysis of sensor kinetics implied the presence of similar adsorption sites in both kinds on functionalised graphenes, indicating that the deposited V_2_O_5_ nanophase was a predominating NH_3_ receptor. The difference in the magnitudes of relative gas response was a result of different charge carrier type and concentration of graphene samples. Epitaxial graphene on SiC was remarkably resistant to PLD plasma. The signals of sensors on SiC/EG platform were larger, faster and more reversible than those for CVD graphene. However, the transferability of CVD graphene onto almost any surface could be of great value.

Graphene is almost ideally suited as a signal transducer material in conductive sensors. Since pristine graphene is a relatively weak and non-specific adsorbent, the progress will essentially depend on the quality of receptor function created by means of functionalisation. Only time will tell whether graphene is superior to other categories of thin conductors, such as nanoSMOX (semiconducting metal oxides) and TMD (transition metal dichalcogenides) [54,55,56].

## Figures and Tables

**Table 1:** Bi-exponential fitting parameters of functionalised CVD graphene sensor response and recovery curves at 8 ppm NH_3_, as shown in Figure 4 and Figure 5, with Equation (1). The coefficients *t*_1_ and *t*_2_ are time constants in the exponential functions. Response amplitude (*A*_1_ + *A*_2_)/*G*_0_ is shown in %. RH stands for relative humidity.

CVD Graphene on Si/SiO_2_		Response			Recovery	
Line No.	Sample/Conditions	*t*_1_ (s)	*t*_2_ (s)	Response (%)	*t*_1_ (s)	*t*_2_ (s)
1	Pristine, UV, RH20	79	1887	−4.7	1607	-
2	V_2_O_5_, no UV, RH20	25	363	−18	47	466
3	V_2_O_5_, UV, RH0	23	329	−16	43	640
4	V_2_O_5_, UV, RH20	21	226	−22	41	427
5	V_2_O_5_, UV, RH50	19	158	−25	62	478

**Table 2:** Bi-exponential fitting parameters of functionalised epitaxial graphene sensor response and recovery curves at 8 ppm NH_3_, shown in Figure 4 and Figure 5, with Equation (1). The coefficients *t*_1_ and *t*_2_ are time constants in exponential functions. Response amplitude (*A*_1_ + *A*_2_)/*G*_0_ is shown in %. RH stands for relative humidity.

Epitaxial Graphene on SiC		Response			Recovery	
Line No.	Sample/Conditions	*t*_1_ (s)	*t*_2_ (s)	Response (%)	*t*_1_ (s)	*t*_2_ (s)
1	Pristine, UV, RH20	143	695	50	658	-
2	V_2_O_5_, no UV, RH20	16	443	−39	56	610
3	V_2_O_5_, UV, RH0	23	474	131	41	496
4	V_2_O_5_, UV, RH20	19	271	160	30	392
5	V_2_O_5_, UV, RH50	26	182	216	57	440
